# P-2211. 5-Aminosalicylic Acid Prevents Antibiotic Induced Expansion of *Candida albicans* in the Gastrointestinal Tract

**DOI:** 10.1093/ofid/ofae631.2365

**Published:** 2025-01-29

**Authors:** Derek J Bays, Hannah Savage, Connor Tiffany, Mariela Gonzalez, Eli Bejarano, Thaynara Carvalho, Zheng Luo, Hugo Masson, Henry Nguyen, Renato Santos, Krystle Reagan, George R Thompson, Andreas Baumler

**Affiliations:** UC Davis Health, Sacramento, California; UC Davis Veterinary School of Medicine, Davis, California; CHOP, Philadelphia, Pennsylvania; UC Davis, Davis, California; UC Davis, Davis, California; UC Davis, Davis, California; UC Davis, Davis, California; UC Davis, Davis, California; UC Davis, Davis, California; Universidade Federal de Minas Gerais, Davis, California; UC Davis School of Veterinary Medicine, Davis, California; University of California Davis Medical Center, Sacramento, CA; UC Davis, Davis, California

## Abstract

**Background:**

Antibiotic treatment sets the stage for intestinal domination by *Candida albicans* which is necessary for development of invasive disease, but the resources driving this bloom remain poorly defined. We sought to determine these factors in order to design novel prophylaxis strategies for reducing gastrointestinal (GI) colonization.

Figure 1

**Figure:**
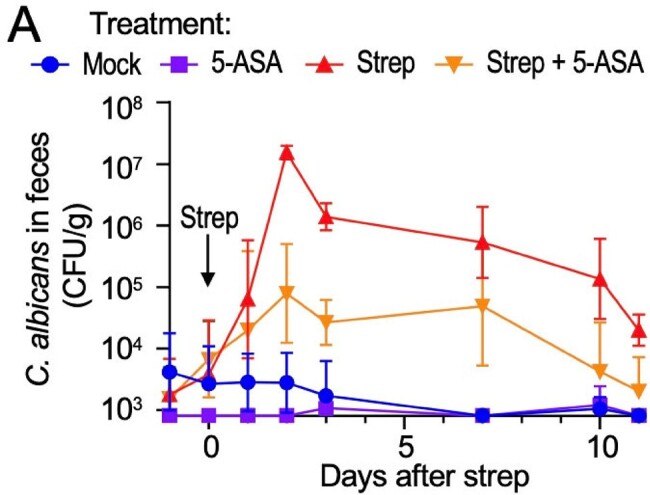

C. albicans colonization (CFU/g feces). All mice were treated with 10^6 of C. albicans (ATCC 28367) and randomized to receive mock treatment, 0.25% 5-ASA in mouse chow, streptomycin 20mg via oral gavage, and 0.25% 5-ASA in mouse chow with streptomycin 20mg via oral gavage. Feces was collected to enumerate C. albicans colonization.

**Methods:**

We initially developed a generalizable framework, termed metabolic footprinting to determine the metabolites *C. albicans* preferentially uses in the mouse GI tract. After identifying the metabolites *C. albicans* utilizes, we used in vitro growth assays in the presence and absence of oxygen to validate out metabolomics findings. We next determined if a probiotic *E. coli* that utilizes oxygen would reduce *C. albicans* colonization compared to a mutant *E. coli* that could not respire oxygen. Finding that oxygen was a necessary resource, we utilized germ-free mice to determine if *Clostridia* spp. known to reduce GI oxygen would prevent *C. albicans* colonization. Lastly, we sought to see if 5-aminosalicylic acid (5-ASA) could prevent *C. albicans* colonization.

Figure 2

**Figure:**
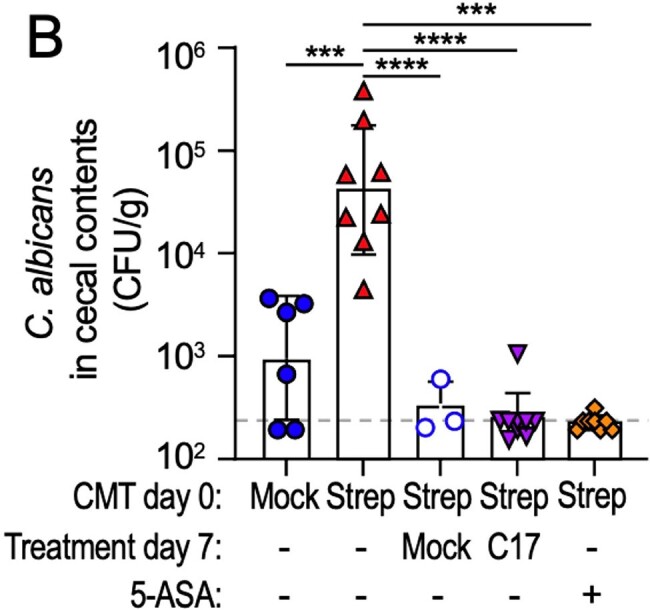

Germ-free Swiss Webster mice received a cecal microbiota transplant from streptomycin-treated C57BL/6J mice (Strep) or from mock-treated C57BL/6J mice (Mock). Seven days later, some mice were inoculated with a community of human Clostridia isolates (C17), received a second cecal microbiota transplant from mock-treated C57BL/6J mice (Mock), or were switched to chow supplemented with 5-aminosalicylic acid (5-ASA: +). Seven days later, all mice were challenged with 105 C. albicans CFU (strain ATCC28367) and cecal contents were collected 1 week later to enumerate C. albicans CFU/g cecal contents

**Results:**

We found that *C. albicans* preferentially utilizes simple carbohydrates including fructo-oligosaccharides (e.g., 1-kestose), disaccharides (e.g., β-gentiobiose), and alcoholic sugars (e.g., sorbitol) and is able to grow in vitro on minimal media supplemented with either of these nutrients. However, in the hypoxic environment that is found in the "healthy" colon, *C. albicans* cannot utilize these nutrients. We next found that pre-colonization in a mouse model with a probiotic *E. coli* significantly reduced *C. albicans* colonization, but the mutant *E. coli* had no effect on colonization. We next showed that Clostridia supplementation restored GI hypoxia and reduced *C. albicans* colonization. Remarkably, we found that 5-ASA significantly reduced GI colonization of *C. albicans.*

**Conclusion:**

We have shown that *C. albicans* requires oxygen to colonize the GI tract. Importantly, we found that 5-ASA can prevent an antibiotic mediated bloom of *C. albicans* by restoring GI hypoxia, which warrants additional studies to determine if 5-ASA can be used as an adjunctive prophylactic treatment in high-risk patients.

**Disclosures:**

George R. Thompson, III, MD, Astellas: Advisor/Consultant|Cidara: Advisor/Consultant|Cidara: Grant/Research Support|F2G: Advisor/Consultant|F2G: Grant/Research Support|Melinta: Advisor/Consultant|Melinta: Grant/Research Support|Mundipharma: Advisor/Consultant|Mundipharma: Grant/Research Support|Pfizer: Advisor/Consultant

